# Prediction models in prostate cancer: a systematic review and meta-analysis

**DOI:** 10.3389/fonc.2026.1705780

**Published:** 2026-05-01

**Authors:** Emmanuel Nsedu Israel, Kaze N. Niels, Omobolanle Oluwatosin Makanjuola, Oluwakemi A. Rotimi, Israel S. Afolabi

**Affiliations:** 1 Department of Biochemistry, College of Science and Technology, Covenant University, Ota, Ogun, Nigeria; 2 Covenant Applied Informatics and Communication - Africa Centre of Excellence (CApIC-ACE), Covenant University, Ota, Ogun, Nigeria

**Keywords:** deep learning, machine learning, meta-analysis, prediction models, prostate cancer, systematic review

## Abstract

**Background:**

Prostate cancer is the sixth most common cause of cancer-related death in males globally and is often identified as a secondary malignancy. Early detection and appropriate management increase the patient’s life expectancy. In predicting prostate cancer outcomes, artificial intelligence models are currently employed increasingly, although they are not widely applicable due to an unclear understanding of their performances. This systematic review and meta-analysis examined the pooled predictive performances of these models using area under the curve (AUC) metrics across different prediction endpoints: overall survival (OS), progression-free survival (PFS), toxicity or quality of life (TQL), recurrence distance metastasis (RDM), and treatment response (TR).

**Methods:**

PubMed and Scopus were systematically searched for studies that reported prediction models for prostate cancer outcomes. In accordance with PRISMA criteria, we carried out a systematic review of prediction models for prostate cancer, with a registered protocol in PROSPERO (CRD42025611480). Using the Prediction Model Quality Score (PMQS), qualified studies were assessed for methodological quality. We conducted a random-effects meta-analysis, pooling AUCs for each prediction endpoint; heterogeneity was assessed using I², τ², and Q statistics. Funnel plots, Egger’s regression test, and the trim-and-fill method were used to assess publication bias.

**Results:**

A total of 144 studies were selected based on the eligibility criteria for systematic review, but only 85 were included in the meta-analysis. The overall pooled AUC in all prediction endpoints were 0.808, 0.792, 0.845, 0.835, and 0.805 for OS, PFS, RDM, TR, and TQL, respectively, indicating good predictive performance. Funnel plots showed no significant asymmetry.

**Conclusion:**

The models evaluated in this review demonstrated over 75% accuracy in predicting prostate cancer with varied performance across prediction endpoints. Standardized model reporting and robust external validation are needed to improve reproducibility and clinical translation.

**Systematic Review Registration:**

https://www.crd.york.ac.uk/PROSPERO/view/CRD42025611480, identifier CRD42025611480.

## 1 Introduction

One of the biggest health issues affecting men is prostate cancer (PCa). Prostate cancer ranks sixth globally in terms of cancer-related mortality among males and is often identified as a secondary malignancy. In 2020, there were 1,414,259 incident cases and 375,304 fatalities. Africa has 93,173 reported deaths which is about 6.6% of the total recorded number (1).

At the early stages prostate cancer is asymptomatic, but at later stages some symptoms like blood in urine, frequent urination, difficulty in urination and holding back urine, and painful urination may occur (1). When any of these symptoms are observed, the person may be at risk or probably have developed prostate cancer. Therefore, some further procedures or tests are usually done to confirm the state of the prostate. They include, tests for prostate-specific antigen level, digital rectal examination, prostate biopsy, multiparametric magnetic resonance imaging (mpMRI), and Prostate-specific membrane antigen positron emission (PSMA PET) (2).

Over the past few decades, as medical informatics and technology have advanced, a significant amount of data about cancer therapy has been produced and maintained globally in (un)structured electronic formats (3). Owing to the useful and practical information they may offer about a patient’s course of therapy and results, the application of machine learning is speedily taking the lead in cancer therapy and management (4, 5). These insights may aid carers in choosing the best course of action for a patient or in adjusting their current course of treatment in order to enhance the patient’s result (6). This is crucial, especially for high-risk patients who don’t respond well to conventional therapy methods (7).

In recent years, routine clinical imaging has created a gold mine for machine learning. Imaging data is among the references used in oncology machine learning (8). In particular, Magnetic Resonance Imaging (MRI) and Positron Emission Tomography/Computerized Tomography (PET/CT) are utilized for follow-up exams, pre-treatment staging, diagnosis, and intervention planning (9, 10). Researchers have investigated technologies in machine learning, including auto-contouring and computer-aided diagnosis (CAD) to help with medical picture interpretation and diagnosis (4, 11–13). Additionally, in personalized medicine, where patients are divided into subpopulations according to how well they respond to treatment or how susceptible they are to disease, technologies that use machine learning can locate a patient’s unique tumor data contained in a medical image’s pixels. This information can then be obtained by means of deep learning and radiomic techniques to provide the best possible therapeutic intervention (14). Automating the treatment process, enhancing patient care, and optimizing treatment outcomes for prostate cancer are all possible with machine learning techniques (10).

Over the past ten years, a number of prediction models have been created, evaluated, and applied to cancer screening and therapy for various outcomes of interest (11, 15–18). We conducted a systematic evaluation of publications on prediction models for prostate cancer as part of our investigation. In particular, this review’s major goal is to evaluate prediction accuracy, and its secondary goal is to evaluate the technological advancements in prediction model creation.

## 2 Methods and design

This study employed the Preferred Reporting Items for Systematic Reviews and Meta-Analyses (PRISMA) guidelines, with the process summarized in a PRISMA flow diagram. The research question was frame using PICOS framework: Population: prostate cancer patients, Intervention: machine learning and deep learning prediction model, Comparators: methodological quality and significance based on existing approaches, Outcomes: prostate cancer endpoints such as recurrence or distant metastases, overall survival, progression-free survival, response to treatment, toxicity, or quality of life, Study design: original research published in peer-reviewed journals. To examine the meta-analysis of prediction models in prostate cancer, the study adopted and modified the search procedures outlined by Kitchenham (19) and Brocke et al. (20). This study explored the following research questions: What are the feature selection techniques, common features, and prediction endpoints used for training prostate cancer prediction models? How effective is machine learning in accurately predicting prostate cancer endpoints such as recurrence or distant metastases, overall survival, progression-free survival, response to treatment, toxicity, or quality of life? How do prostate cancer prediction models compare in terms of methodological quality and significance based on existing approaches?

### 2.1 Protocol registration

This review protocol has been registered in PROSPERO with the identification number CRD42025611480.

### 2.2 Eligibility criteria

All original papers finally chosen for this review fulfilled the following eligibility criteria: confirmed prostate cancer cases for any histology group, developed a model and included prediction endpoints for models developed, and published articles exclusively in English between 2012 and 2024.

### 2.3 Exclusion criteria

We removed articles based on the following criteria: non-human subjects as samples, prognosis and prediction models were not developed, and tested, only gene sequence data was used for model development.

### 2.4 Information source and search method

We searched through Scopus and PubMed databases for relevant publications on October 17, 2024. The study constructed search keywords, which were used to execute search queries to identify relevant articles from the databases between 2012-2024. The search criteria include extraction of primary keywords from the research questions, construction of synonyms from the extracted keywords, identification of relevant keywords from the extracted articles, and connection of keywords with the use of Boolean operators such as “AND” or “OR.” The search keywords used to execute search queries to retrieve relevant articles include, prostate cancer, prostate tumors, risk prediction models and prediction models. A full search strategy is provided in supplementary **Supplementary Table S3**.

### 2.5 Study selection and data extraction

Guided by the inclusion criteria abstracts and eventually the full articles were screened independently by two authors (O.M and K.N), and validated by a third author (E.I.N.). Eligible articles were downloaded, and data were extracted, saved, and preprocessed in an Excel file. Among the data that was extracted were the authors, publication year, size of sample, cohort characteristics, feature used, disease stage, treatment, endpoint to prediction, model used, model assessment, accuracy of the best model in the validation set, feature selection technique, statistical test used to evaluate model performance, and validation (internal or external).

### 2.6 Data analysis

The selected studies were grouped into five clinical endpoints that were used to build the models. These groups are group 1: overall survival, group 2: progression-free survival, group 3: recurrence or distant metastasis, group 4: toxicity or quality of life, and group 5: treatment response. For each group, model performance was evaluated based on their performance in their respective groups.

### 2.7 Risk of bias assessment and methodological quality

To assess the methodological quality and risk of bias of the included prediction model studies, we used the Prediction Model Quality Score (PMQS) and Prediction Model Risk of Bias Assessment Tool (PROBAST) (21). PROBAST uses four metrics to evaluate risk of bias, which includes participants, predictors, outcome, and analysis. Two reviewers (K.N. and O.M.) independently applied PROBAST to all eligible studies. All disagreements were resolved by involving a third reviewer (E.I.N.).

Each study was categorized as low, high, or unclear risk of bias, following PROBAST guidance. Since Radiomic Quality Score (RQS) only assesses radiomic-based prediction models, while Prediction Models for Individual Prognosis or Diagnosis (TRIPOD) assesses total adherence to both development and reporting of prediction models, we employed the PMQS scoring system, which was proposed by A.K. Jah et al. (14), to assess the quality of studies on prediction models based solely on development and validation. Studies are classified as most significant, significant, moderately significant, and least significant with scores ranking > 60%, > 50%, > 40%, and < 40%, respectively, according to the PMQS scoring system shown in **Table 1**.

**Table 1:** The PMQS scoring system.

Item no	Item	Description	Scoring criterion	Maximum possible score
1	Sample size	One important factor in creating a prediction model is the sample size. Only a prediction model that has been trained on a sizable sample size can effectively capture data diversity and perform well on both external and prospective data.	SS ≤ 100 = 1 100 < SS ≤ 200 = 2 200 < SS ≤ 500 = 3 500 < SS ≤ 1000 = 4 SS > 1000 = 5	5
2	Disease Stage	The methodology makes reference to the disease’s stage.	No = 0 Yes = 1	1
4	Treatment delivered	Treatment has an impact on the disease’s prognosis as well; therefore, it can also be a crucial component in understanding how well the prediction model performs.	No = 0 Yes = 1	1
5	Features used	Numerous studies have found that prediction models with different types of parameters produce superior prediction results.	Only clinical =1 Clinical & demographic = 2 Pathological only =1 Imaging only = 1 Radiomics only = 1	6
6	Event rate reported	In order to determine the skewness of the data.	No = 0 Yes = 1	1
7	Multi-center study	Since the multi-center study gives the prediction model generalizability, it is a crucial factor to take into account when rating the prediction model.	No = 0; Yes = 2 (two centers), 3 (three and more centers), 4 (≥3 centers with various countries)	4
8	Feature selection Technique	Feature selection is crucial, and the method must be explained. A robust model could be developed as a result of a strong feature selection.	Not described =0 Only one method was used =1 Multiple methods used = 2	2
9	Prediction Algorithm Tested	To choose the optimal algorithm for a certain prediction endpoint, one must test a number of algorithms, as one may not be appropriate for all types of prediction problems.	One algorithm one model =1, One algorithm with multiple models = 2, Numerous algorithms with multiple models = 3	3
10	Model Assessment	Understanding the model’s overfitting requires knowing its training and validation scores.	Only validation accuracy without a confidence interval (CI)= 1, validation accuracy with CI = 2, train and validation accuracy with CI = 3	3
11	Model Validation	One of the most crucial elements of model development is the validation method for prediction models. Because external validation guarantees the consistency of the prediction model created, it receives a higher rating than internal validation.	Train-test model validation =1 Bootstrap validation/cross validation = 2 External validation = 4	4

### 2.8 Statistical analysis of model performance

We carried out a meta-analysis in R 4.4.1 (22), utilizing a random-effects model for the various endpoints for prediction. Since AUC (Area Under the Curve) offers a reliable assessment of a model’s capacity for class discrimination and is threshold-independent, it was chosen as the main performance indicator. The AUC and confidence intervals (CI) of the models at validation were retrieved and logit transformed, from which the standard errors were derived. The inverse variance method was used to summarize the AUC. It was calculated with constrained highest probability and adjusted for Knapp and Hartung to produce 95% confidence intervals. Heterogeneity was also examined using Cochran’s Q-test (p < 0.10 considered significant, and I^2^ statistics (low: <25%, moderate: 25-50, and high: >50% (22). Tau-squared (t^2^), estimating between-study variance, was also done, as well as meta-regression to identify the source of heterogeneity.

To evaluate the robustness of the pooled estimate, a leave-one-out sensitivity analysis was conducted based on prediction endpoints, using the DerSimonian–Laird method (23). Each study was sequentially removed, and the meta-analysis was re-run to assess its influence on the overall effect size. Each prediction endpoint’s forest plot was created independently. We used two complementary techniques, the trim-and-fill approach and Egger’s regression test for funnel plot asymmetry, to evaluate the risk of publication bias. By regressing standardized effect sizes on their standard errors, Egger’s test assesses small-study effects. A substantial intercept raises the possibility of publishing bias and asymmetry. By imputing these studies, the trim-and-fill strategy modifies the pooled effect size to account for the estimated number of potentially missing studies (caused by publication bias). The metafor R package’s trimfill() and regtest() functions were used to implement both approaches. Every prediction endpoint underwent a separate analysis.

## 3 Results

### 3.1 Selection of studies

**Figure 1:**
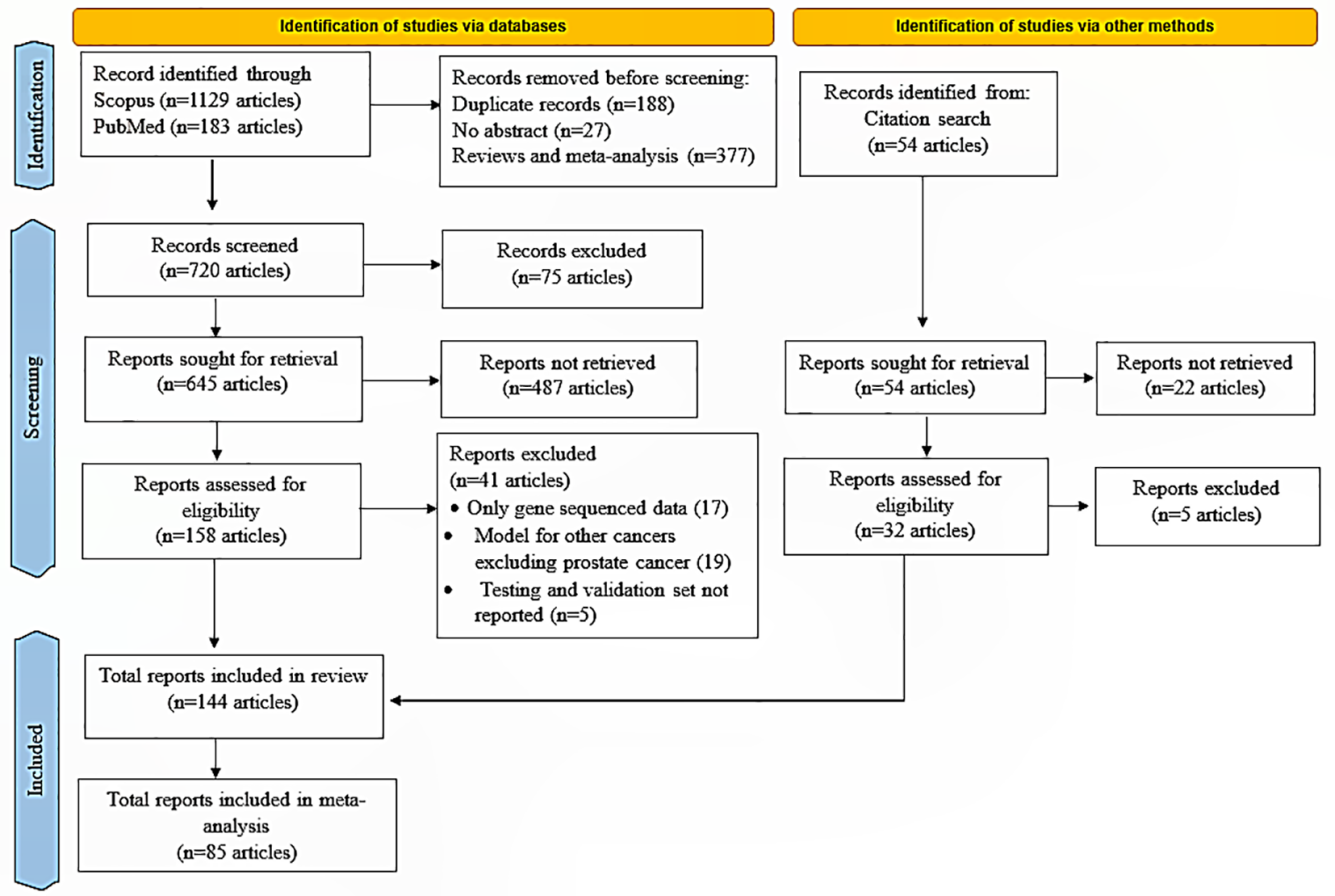
PRISMA workflow.

A total of 1,129 articles were retrieved from Scopus, 183 articles from PubMed, and 54 articles from other sources. Out of 190 articles that were qualified for inclusion, 46 were excluded due to insufficient data presentation, including prediction endpoints and AUC values. The chosen article’s complete manuscripts were downloaded, and extracted data were further analyzed. The sample sizes for all retrospective research that was part of this review were 11 to 283,252. Also, in this review, all stages of prostate cancer were reported, from early stage to advanced castration-metastatic prostate cancer (I-IV). The 144 selected studies include 26 studies for overall survival as an outcome, 17 studies for progression-free survival, 66 studies for recurrences or distant metastasis, 11 studies for toxicity or quality of life, and 24 studies for treatment response. Other data are available in supplementary **Supplementary Table S1**. The selection flowchart of these articles is shown in **Figure 1**, the preferred reporting items for systematic reviews and meta-analyses (PRISMA) (24).

### 3.2 Risk of bias assessment and methodological quality

We used the PROBAST tool to assess the risk of bias and PMQS for methodological quality across all included studies (n = 144). PROBAST showed that 65.7%, 23.6%, and 10.4% of the studies were rated as low, unclear, and high risk. For the specific domain, 86% had a low ROB in participants, although some studies used single-center and retrospective data, which may limit generalizability. Predictors were clearly defined and assessed prior to outcome in the majority of the studies, while some studies had no justification for predictor inclusion. Generally, outcomes were appropriate, and while analysis also had a low ROB, it was observed that the majority of the studies lacked external validation. The ROB and applicability chart is shown in supplementary **Supplementary Figure 1**.

Furthermore, the methodological quality and significance of the identified studies address the third research question. All included studies were assessed and scored based on the PMQS system, in which 88.9% of the included studies were rated moderately to most significant. Notably, the studies by Wang, X et al. (25), Muehlematter, U et al. (26), and Yang, J et al. (27), were categorized as highly significant, significant, and least significant, respectively. This PMQS report is summarized **in Supplementary Table 2**.

### 3.3 Feature selection techniques, common features and prediction endpoints used for training prostate cancer prediction models

This section addresses the first research question, which explores the feature selection techniques, common features, and prediction endpoints employed in developing prostate cancer prediction models. The study identified various machine learning models that utilized clinical, demographic, pathological, imaging, and radiomic features to predict prostate cancer outcomes.

The study identified several feature selection approaches to select important features to ensure optimal performance of the machine learning prostate cancer diagnosis model, including univariate logistic regression, univariate Cox regression, Kaplan-Meier analysis, Pearson or Spearman correlation, and Shapley Additive Explanation (SHAP). These techniques were commonly applied in the identified articles to select the most relevant features for training the models. Further details of these methods are provided in **Supplementary Table 1**, showing their varied application across studies.

The integration of clinical, imaging, and pathological data was common across several studies. For example, Isaksson et al. (28) developed prostate cancer prediction models using clinical data such as age, PSA level, and comorbidities, alongside imaging features like prostate volume, the number of dominant lesions, and the PI-RADSv2 score. These features were used to predict endpoints such as postoperative ISUP grade group, pathological tumor stage (T), lymph node status (N), surgical margin status, and both biochemical and clinical progression. Similarly, Lee et al. (29) utilized clinical features, MRI PRECISE scores, and biopsy results to predict disease progression, including upgrading to higher-grade disease and progression to more advanced stages.

In addition, Hou et al. (30) used MRI radiomics signatures alongside clinical MRI and histopathological data. The primary endpoint for this study was biochemical recurrence-free survival, which was influenced by the MRI radiomics features. Wang et al. (31) analyzed a dataset comprising 2,716 nmCRPC patients. The training dataset was retrieved from a Phase III clinical trial, SPARTAN, while the testing dataset came from the ARAMIS trial. The metastasis-free survival was used as the primary endpoint, and the authors evaluated thirteen clinical features, including baseline PSA level, Gleason score, PSADT, race, and previous treatments received, using ten machine learning models to predict metastasis.

Other studies, such as Peng et al. (32), proposed machine learning prognostic models for the overall survival of prostate cancer patients with lymph node-positive using demographic and clinical data from the SEER database. The common clinical features included age, clinical T stage, PSA level, Gleason Score (GS), and number of positive lymph nodes. The data also included variables such as radical prostatectomy (RP), radiotherapy (RT), and follow-up information, with stepwise selection used to identify prognostic variables. The primary endpoint was overall survival (OS), calculated from diagnosis to death.

The most common prediction endpoints identified in this study include biochemical recurrence, metastasis-free survival, overall survival, and disease progression (such as upgrading to higher Gleason scores or advancing to more severe tumor stages). This suggests that these endpoints are significant in assessing machine learning algorithms’ predictive performance in prostate cancer prediction, and they also align with meaningful clinical outcomes.

### 3.4 Machine learning algorithms for prostate cancer prediction

This section addresses the second research question, which investigates the machine learning algorithms used in prostate cancer prediction. A variety of prediction algorithms have been employed in the identified articles, all demonstrating reasonable predictive performance, as indicated by an area under the curve (AUC) or concordance index (C-index) greater than 0.7 in validation. The algorithms commonly used across these articles include Random Forest (RF), multivariate logistic regression (MLR), Support Vector Machines (SVM), the multivariate Cox proportional hazards model (Cox-mPH), and Extreme Gradient Boosting (XGBoost).

Additionally, deep learning models, including Convolutional Neural Networks (CNN), Dense Neural Networks (DNN), and Recurrent Neural Networks (RNN), were also applied. Other algorithms such as K-Nearest Neighbors (KNN), Probabilistic Neural Networks (PNN), and Gradient Boosting Machines (GBM) were utilized in some studies to enhance prediction accuracy. Artificial Neural Networks (ANN) and Survival Trees were also employed, further demonstrating the diversity of approaches to predicting prostate cancer outcomes.

In terms of specific algorithms, logistic regression emerged as one of the most widely used algorithms, with thirty-three studies employing it to predict various outcomes in prostate cancer patients. Among these studies, logistic regression was frequently used to predict recurrence or distant metastasis (25, 33–56), with a smaller number focusing on progression-free survival (57–59) or treatment response (60–63). Notably, only one study used logistic regression for predicting overall survival (64), and none employed it for toxicity or quality of life outcomes. This demonstrates the effectiveness and predominance of logistic regression in predicting cancer recurrence or metastasis.

Neural networks, on the other hand, were employed in eleven studies, where they were used to predict different prediction endpoints. CNN and DNN were utilized in predicting progression-free survival (65, 66), while ANN and RCN were applied to predict recurrence or distant metastasis (67, 68). These deep learning models demonstrated strong predictive capabilities, reflecting the growing trend of using more complex algorithms to capture intricate patterns in medical data.

Support Vector Machines (SVM) were employed in fourteen studies, though none of these focused on predicting overall survival or toxicity/quality of life. Most studies using SVM focused on predicting recurrence or distant metastasis (41, 42, 49, 50, 69–71), with a smaller number addressing progression-free survival (58, 65, 72) and treatment response (73–76). This suggests that SVM is particularly well-suited for survival analysis and recurrence prediction in prostate cancer.

In addition, decision support tools such as nomograms were used in fifty-seven studies to represent predictive models. These nomograms were primarily employed to predict recurrence or distant metastasis, but they also proved effective for predicting other endpoints, such as overall survival, treatment response, and progression-free survival. All of the nomogram-based models demonstrated high predictive performance (c-index/AUC > 0.7), further emphasizing the value of these tools in clinical decision-making.

### 3.5 Meta-analysis

**Figure 2:**
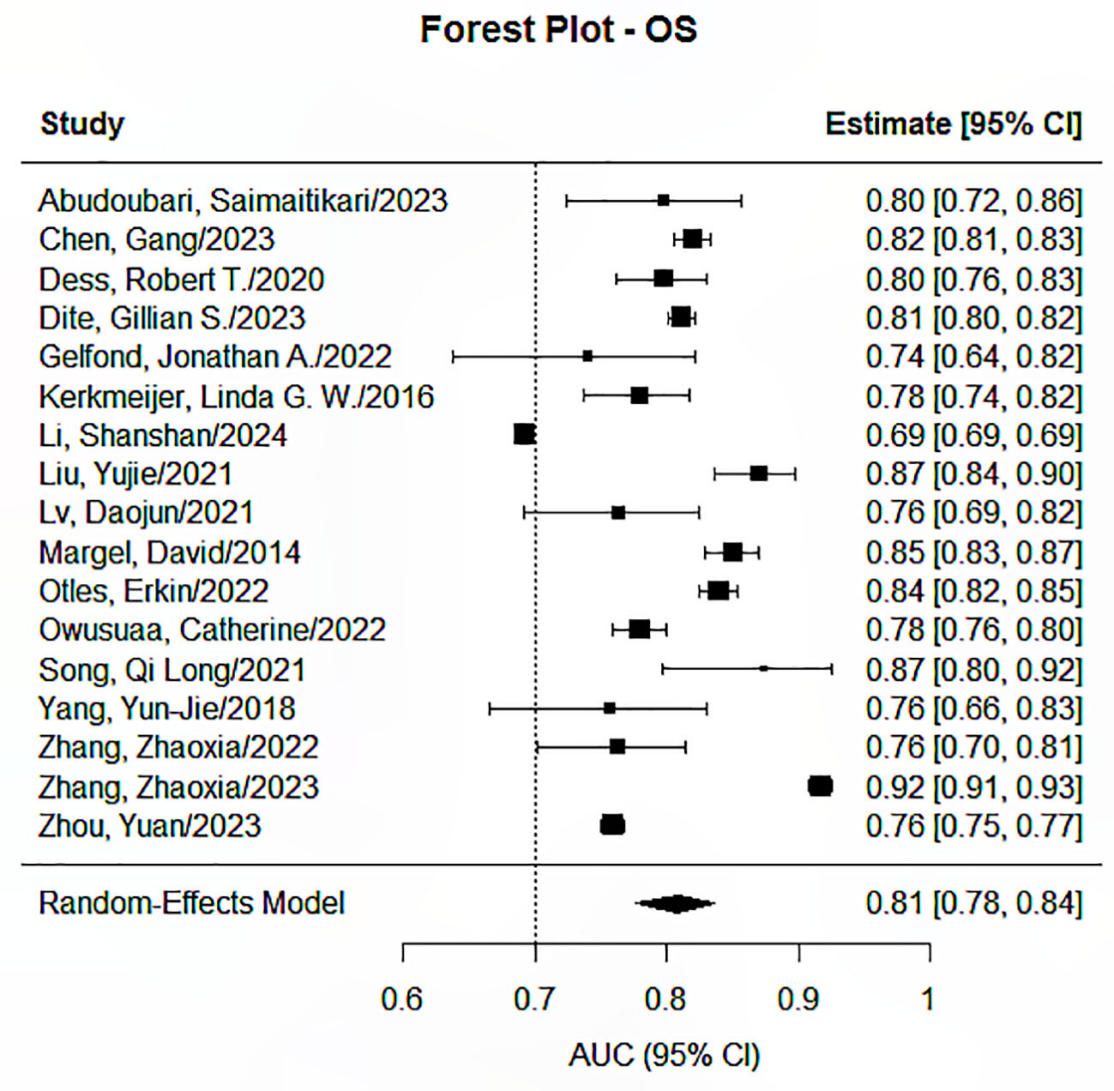
Forest plot for the prediction endpoints (OS).

**Figure 3:**
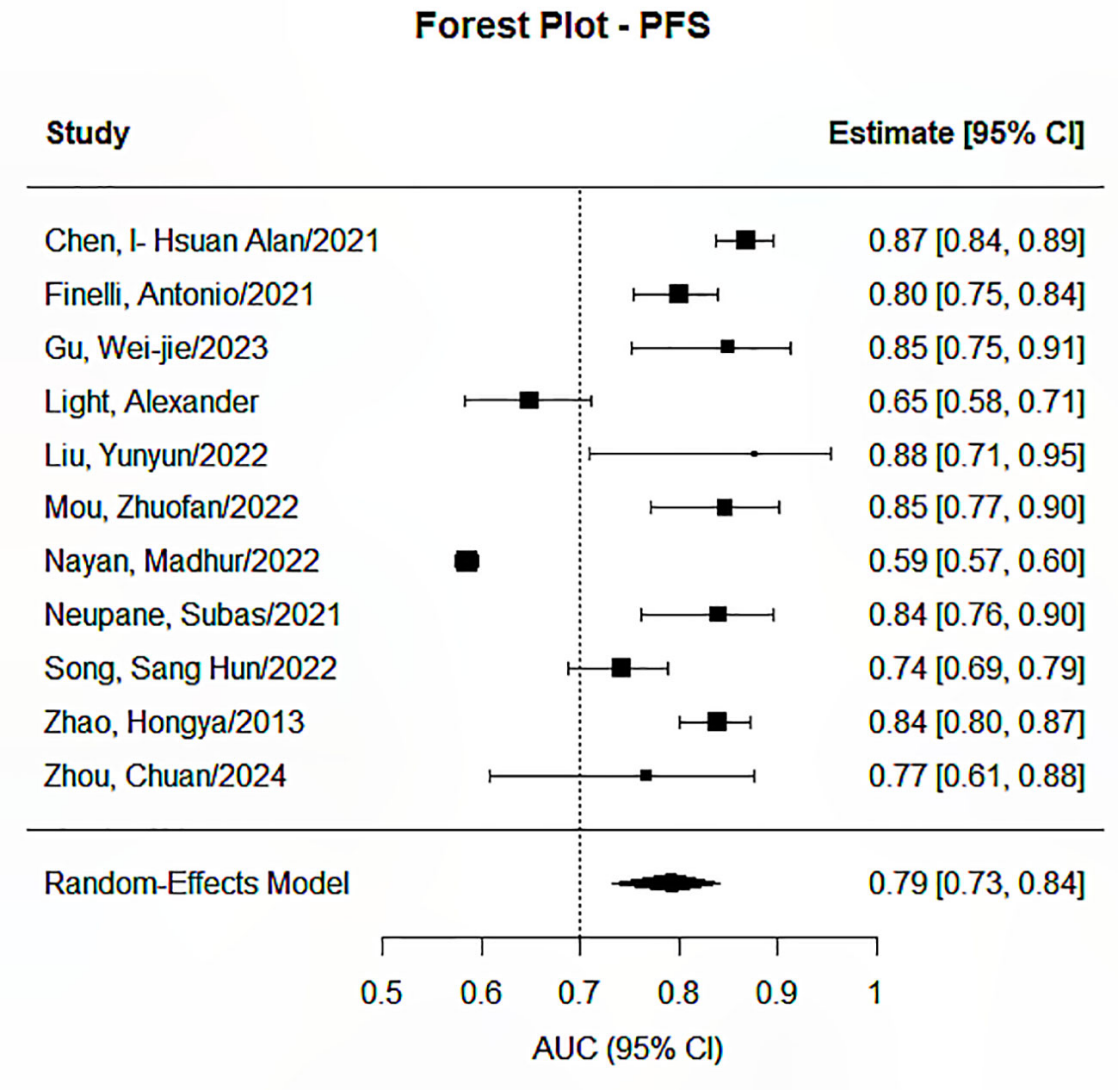
Forest plot for the prediction endpoints (PFS).

**Figure 4:**
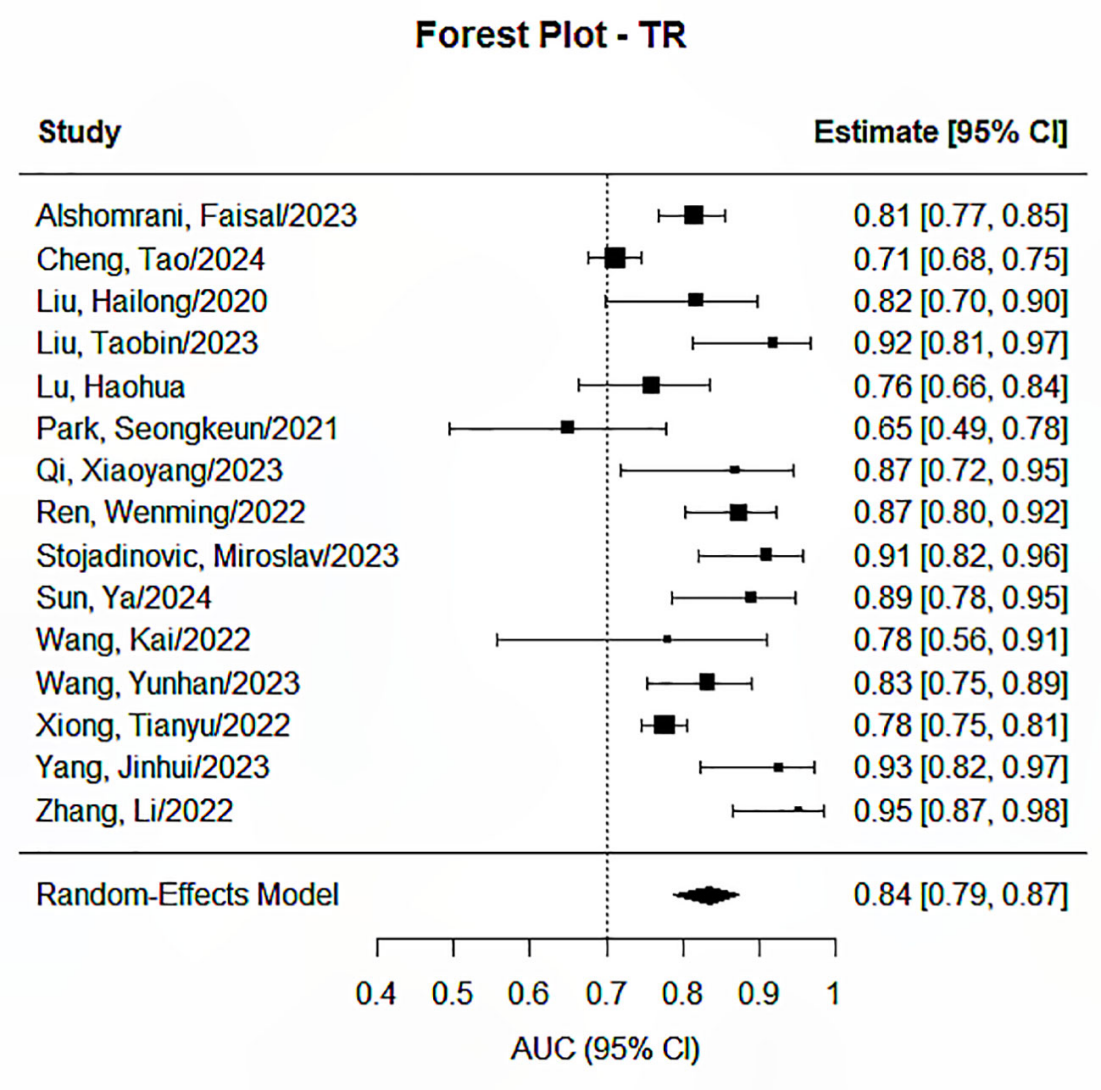
Forest plot for the prediction endpoints (TR).

**Figure 5:**
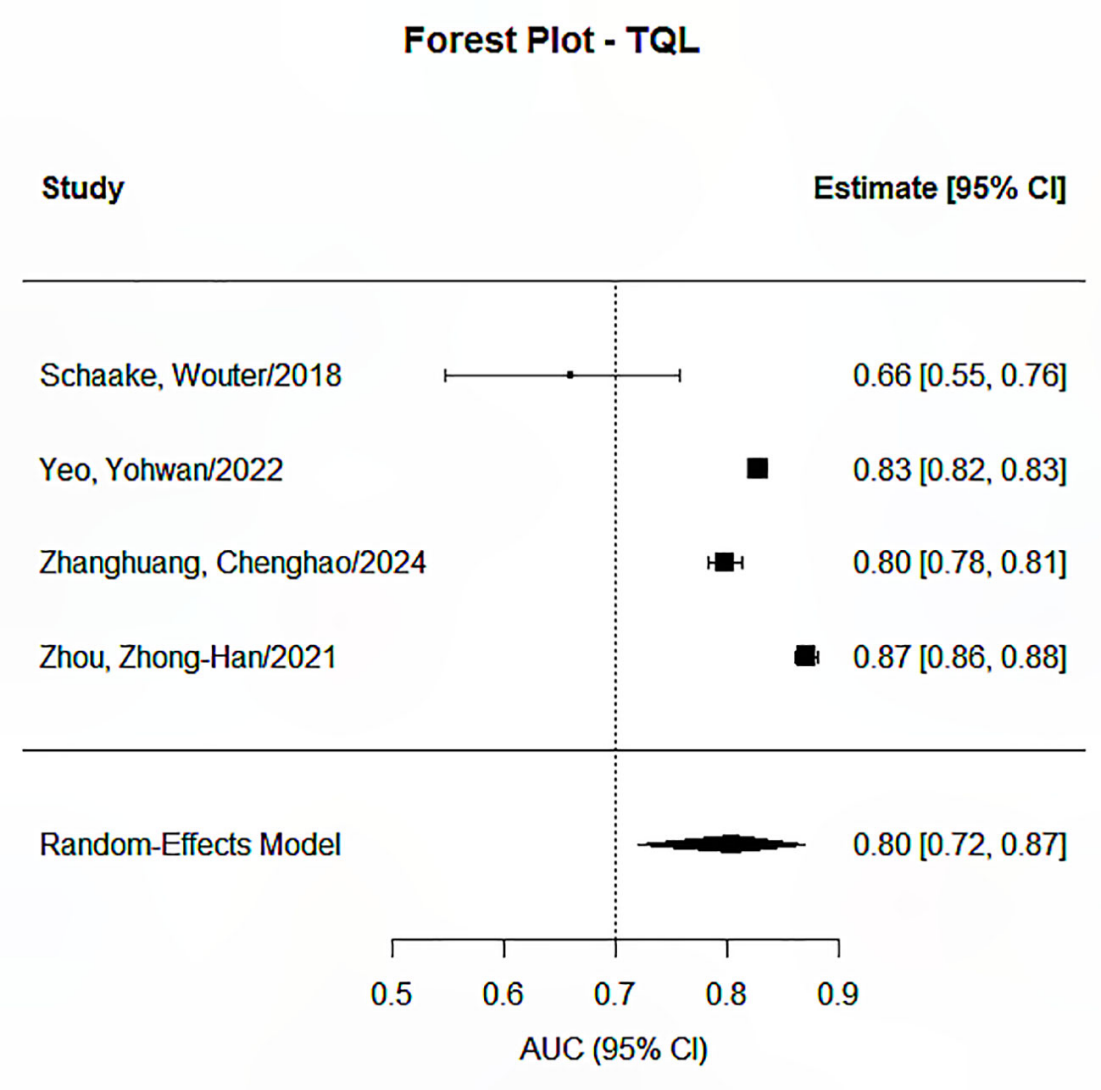
Forest plot for the prediction endpoints (TQL).

**Figure 6:**
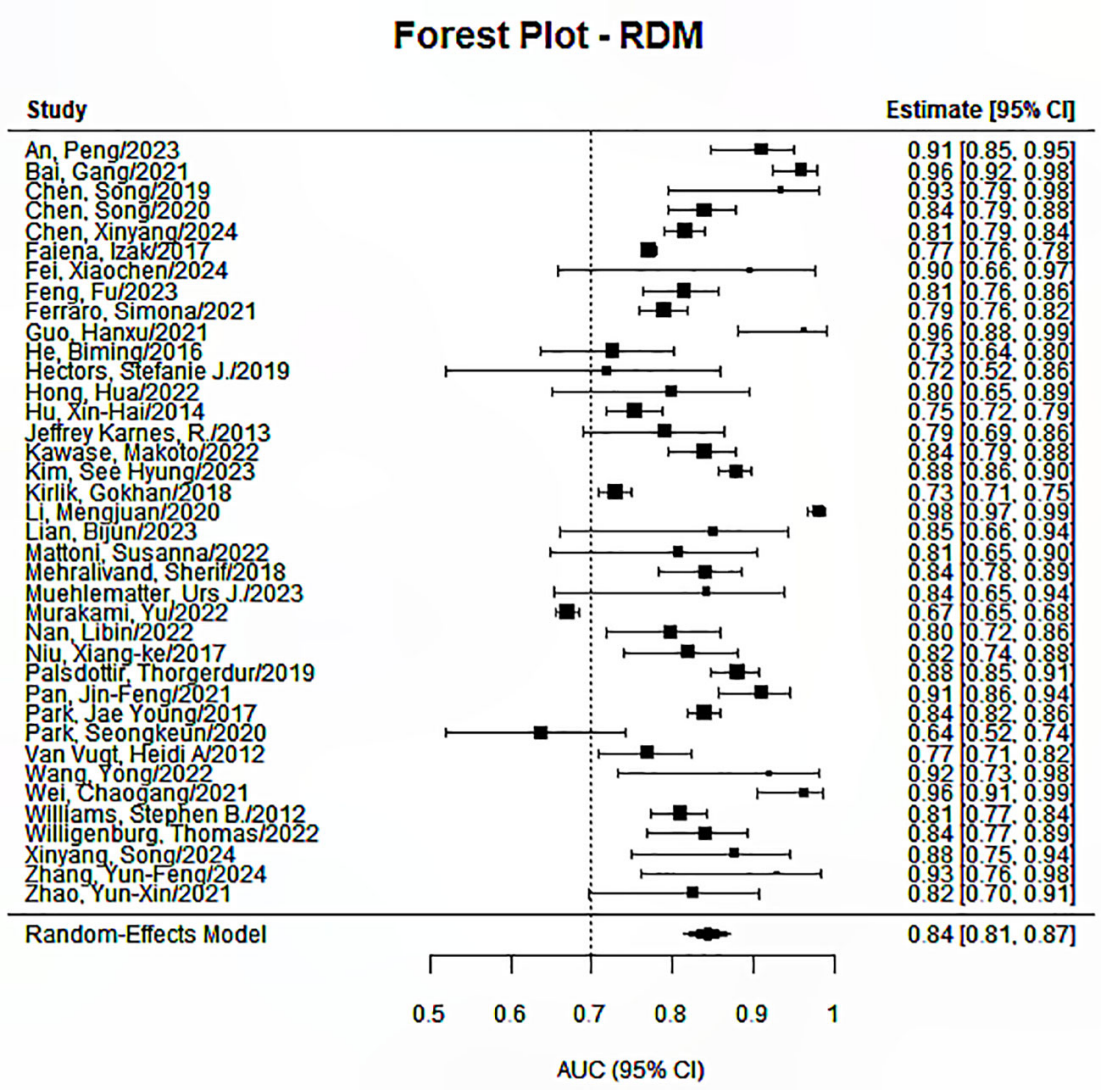
Forest plot for the prediction endpoints (RDM).

The Random Effects Model was applied in conducting the meta-analysis for each prediction endpoint, having envisaged heterogeneity due to the different features and feature selection techniques used, as well as the different models developed. Our research identified significant pooled statistics in all five endpoints, including progression-free survival, overall survival, distant metastasis or recurrence, treatment response, and toxicity or quality of life. The pooled AUCs ranged from 0.79 to 0.85 across groups, showing moderate to good overall performance, as presented in **Figures 2**–**6**. Specifically, the RDM group showed the highest pooled AUC of 0.845 (95% CI: 0.813–0.872), while the OS group had a pooled AUC of 0.792 (95% CI: 0.732–0.842). Prediction intervals (PI) reflected between-study variability, with PIs ranging, for example, from 0.661 to 0.901 for the OS group. Measures of heterogeneity were substantial in all groups (I² values from 84.3% to 99.1%), and Tau² estimates indicated considerable between-study variance. The Cochran’s Q-test was significant for all groups (p < 0.001), confirming the presence of statistically significant heterogeneity as reported in **Table 2**.

**Table 2:** Meta-analysis results for each group.

Group	k	Pooled AUC (95% CI)	Prediction interval (PI)	I² (%)	Tau²	Q-test p-value
OS	17	0.808 (0.776, 0.836)	0.661-0.901	98.7	0.1444	<0.0001
PFS	11	0.792 (0.732, 0.842)	0.568-0.917	94.0	0.2648	<0.0001
RDM	38	0.845 (0.813, 0.872)	0.610-0.950	96.9	0.3921	<0.0001
TQL	4	0.805 (0.720, 0.869)	0.595-0.920	99.1	0.2191	<0.0001
TR	15	0.835 (0.789, 0.873)	0.647-0.933	84.3	0.2447	1.67e-08

A mixed-effects meta-regression model was fitted to explore sources of heterogeneity. Multicollinearity diagnostics (VIF) indicated low risk of collinearity among moderators (sample size, treatment involved, feature selection methods, number of centers involved, model used, validation status), supporting the reliability of the meta-regression estimates. The model explained approximately 18% of the between-study variance (R² = 17.94%), indicating that the included moderators account for a meaningful, though limited, portion of heterogeneity. The overall test for moderators was statistically significant (QM = 33.62, p = 0.0398), suggesting that at least one moderator significantly influences effect sizes. Most individual moderators did not reach statistical significance (p > 0.05), except for validation status (estimate = -0.3292, p = 0.0245), indicating that studies with validation datasets showed significantly different (lower) logit-transformed AUC estimates compared to those without validation.

The leave-one-out sensitivity analysis showed that the pooled AUC estimates remained relatively stable regardless of which study was excluded, ranging from 0.77 to 0.84. This indicates that the overall meta-analytic findings are not unduly influenced by any single study, suggesting good robustness of the results, as shown in supplementary **Supplementary Figure 2**.

Egger’s regression test and the trim-and-fill method were carried out to assess publication bias across all prediction endpoints except TQL due to the small number of included studies for this endpoint. The Egger’s test for funnel plot asymmetry in the PFS and OS groups was not statistically significant (z = 0.3873, p = 0.6985) and (z = 0.5194, p = 0.6035), respectively, indicating no evidence of small-study effects. For the TR group, Egger’s regression test indicated potential funnel plot asymmetry (z = 1.97, p = 0.049), suggesting possible small-study effects or publication bias.

**Figure 7:**
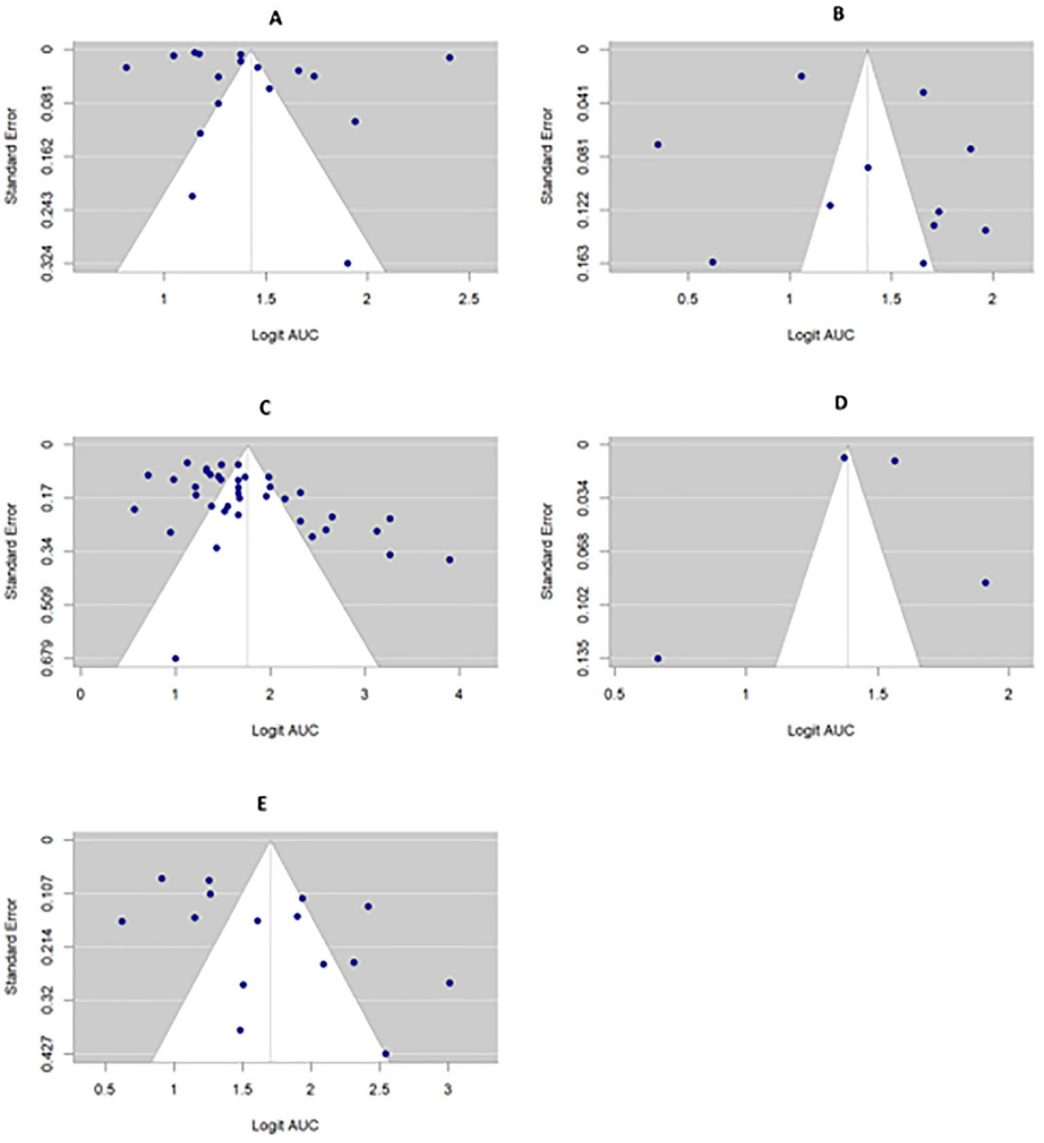
Funnel plot for the prediction endpoints (**(A)**-OS, **(B)** PFS, **(C)** RDM, **(D)** TQL, **(E)** TR).

For the TR and RDM groups, Egger’s regression test indicated potential funnel plot asymmetry (z = 1.97, p = 0.049) and (z = 2.9030, p = 0.0037), respectively, suggesting possible small-study effects or publication bias. However, the trim-and-fill method estimated no missing studies across OS, RDM, and TR endpoints, suggesting that the asymmetry may be due to heterogeneity or other factors rather than true publication bias. For the PFS group, the trim-and-fill method estimated one potentially missing study on the left side; however, this estimate had high uncertainty (SE = 2.2944), and the overall impact on the pooled effect was minimal. Publication bias is presented using a funnel plot in **Figure 7**.

## 4 Discussion

To uncover underlying relationships between characteristics and the output variable, artificial intelligence, specifically, machine learning, uses sophisticated computer and mathematical techniques (77–79). Machine learning has been widely applied in healthcare in improving diagnosis, treatment, and management of different diseases. In prostate cancer, it has been used in predicting major endpoints including diagnosis, disease progression, treatment response, and survival rates (80, 81). Ultimately, the goal of artificial intelligence is to support decision-making for personalized care.

This systematic review and meta-analysis evaluated articles that have developed prediction models for a range of outcomes utilizing clinicopathological, radiological, and radiomics characteristics. For the prediction model’s performance evaluation, the majority of the articles have reported accuracy and area under the curve (AUC), with a few of them carrying out a comprehensive evaluation of the effectiveness of the prediction models. Although, AUC is very important for overall discrimination (82, 83), we acknowledge that AUC alone is not sufficient in fully describing model’s performance (84), but the inconsistency in reporting other metrics such as precision, recall, and F1 across studies limited their inclusion in the meta-analysis. Therefore, we advise generating precision, F1 scores, and recall in addition to the model’s accuracy, especially in imbalanced datasets where they are more informative.

To assess the methodological robustness, we adopted a scoring framework (Prediction Model Quality Score (PMQS)). The suggested grading system thoroughly assesses every facet regarding the model’s development and validation. A 29-point scale and 11 criteria form the basis of the scoring system. Technical concerns pertaining to the development of prediction models have been given top priority and scores, which included prediction algorithm, model validation, model assessment, feature selection technique, multi-center study and sample size. Four of the eleven parameters histology, disease stage, therapy, and the rate of events have lower scores of 0 or 1, and they are all pertaining to the findings’ reporting.

Sample size remains a key limitation. Models trained on small datasets often suffer from overfitting and fail external validation. Although a few studies overcame this limitation by using ensemble or regularization-based algorithms, and some lacked sufficient statistical power or generalizability. This justifies the wider scoring range (1–5) allocated to sample size within the PMQS.

Feature selection is a crucial step in model development. Prediction performance is largely dependent on the features used (85–87). These features include demographic, pathologic, clinical, and radiomic features. In most publications, limited features or variables are employed to build the prediction model, often excluding treatment-related factors, which should be taken into account while building the model, because they could affect the outcome. A recurring finding across the identified studies was that the radiomic features were superior to clinicopathological factors when used alone for prediction. In several studies, a combination of radiomic features and clinicopathological data were utilized, which led to improvements in the performance of prediction algorithms (56, 88–91). This indicates that integrating imaging data with clinical characteristics can provide a more comprehensive approach to prostate cancer prediction.

Based on the impact of features, the best features should be chosen or extracted from the initial set of features. The features’ inclusion criteria ought to be supported by evidence, which includes prior research and/or clinical experience, but only a few studies cited prior literature or clinical expertise to justify choices. Transparent reporting of feature importance and interpretability remains a gap in the field. Thorough explanations of a variety of feature selection methods, including univariate and multivariate analysis have been provided in the literature (92, 93). In order to get rid of less significant features and get beyond problems with overfitting and multi-collinearity, the studies in our review carefully used feature selection techniques. Several authors have chosen the most crucial features for the creation of multivariate models using univariate logistic regression (48, 94–97). However, the procedure for choosing features was not sufficiently evaluated in 47 studies, which limits the transparency and reproducibility of the approaches used.

Another important gap that was observed across the studies included in this review is in stage-specific validation of the models. Unlike some studies that tested their model between disease stages (25, 62, 73), other models trained for early-stage PCa were not tested for predicting late-stage cases and vice versa. This questions the model’s generalizability and clinical relevance across the disease stages. Therefore, it is recommended that future research put this into consideration.

To construct a model, one must choose an acceptable prediction algorithm in addition to the most informative features. Developing and validating a single model with one prediction method is frequently disadvantaged since a given model might not be effective for a given prediction task (77). One benefit of creating several models is that it increases the likelihood that the best model will be identified as one that performs adequately for prediction with the specific features (77). Only a small amount of the research in this review has evaluated and contrasted several prediction algorithms to determine which one best predicts the endpoint.

Another crucial factor that could reveal details about the model’s performance in unidentified datasets is model assessment or validation. Understanding the model’s overfitting requires knowing its training and validation scores. The prediction model is primarily validated using either internal or external data. Numerous validation methods are employed, including bootstrap validation, nested cross validation, cross validation, and train-test validation. Given the significance of the validation stage, PMQS has allocated a score range of 1-4. The prediction model’s generalizability depends on multicenter investigations. Compared to multi-center studies, it is expected that a model created in a single-center study will have a higher likelihood of failing external validation.

Lastly, it is important to emphasize that model development and validation alone are insufficient if the resulting models are not interpretable or clinically relevant. Only a small number of studies included in this review incorporated interpretability methods. While only 1 out of 144 studies applied SHAP (SHapley Additive exPlanations) to identify the most influential variables in their models (98), 34% of included studies did not clearly state what methods were used. A good number of studies (48.6%) reported the use of traditional methods such as univariate and multivariate regression analyses, and 16.0% used least absolute shrinkage and selection operator (LASSO) method. This reveals the significant gap in the adoption and reporting of modern interpretability technique in prostate cancer predictive models. This limitation has critical implications including hindrances in validating models’ behavior, and clinical application. Therefore, to enhance model transparency and clinical applicability, future research should integrate such interpretability techniques during model development.

The pooled results of the random-effect meta-analysis carried out by individual prediction endpoints showed that these models achieve good discriminative accuracy overall, with the pooled AUCs ranging from 0.79 to 0.85. These findings suggest that ML and DL models hold significant promise for outcome prediction in prostate cancer, particularly in supporting personalized treatment decisions. Despite these encouraging results, substantial heterogeneity was observed in all groups (I² values from 84.3% to 99.1%). This heterogeneity indicates a lack of consistency in the creation and validation of prediction models. To further explore the sources of heterogeneity, a meta-regression was conducted using different moderators, including sample size, treatment involved, feature selection methods, number of centers involved, model used, and validation status. Among these only validation status was significant in influencing models’ performance by demonstrating lower AUC estimates, revealing the importance of incorporating validation datasets in model development, which reduces the chances of overfitting and biases (99, 100). Although other moderators were not statistically significant, they are important influencers of heterogeneity. For example, overestimation of models’ performance may be observed in single-center studies compared to multicenter studies given the differences in the diversity of the population and probably overfitting (101).

This review has several limitations. First, only studies reporting AUC were included in the meta-analysis, potentially excluding relevant models evaluated with alternative metrics (e.g., F1-score, precision-recall). Second, we did not stratify models by algorithm type (e.g., tree-based vs. neural networks) due to inconsistent reporting, which may conceal performance differences. Third, although, PubMed and Scopus provided wide coverage of the literature, the inclusion of more databases such as Web of Science and IEEE may have increased coverage. To mitigate this, we conducted a comprehensive manual screening of reference lists of included studies.

## 5 Conclusion

Prediction models will undoubtedly aid in the future management of cancer. According to our review, the application of prediction models in prostate cancer research is on the rise, with over 90% of the included studies published within the last five years, and only about 9% published in the previous decade. The combined PMQS quality score and meta-analysis also point to the prediction model’s important significance in prostate cancer. The integration of these prediction models in electronic health records and a thorough assessment of models, especially in relation to their impact on clinical outcomes, will be important future concerns. Therefore, researchers around the world, especially in Africa, where there is a dearth of genomic dataset (102), should continue to generate relevant data for application in prediction models.

## Data Availability

The original contributions presented in the study are included in the article/**Supplementary Material**. Further inquiries can be directed to the corresponding authors.
