# Supplementary figures and images for: Prediction models in prostate cancer: a systematic review and meta-analysis
Source: Front Oncol. 2026 May 1;16:1705780. doi: 10.3389/fonc.2026.1705780 (PMC13175879; doi:10.3389/fonc.2026.1705780)

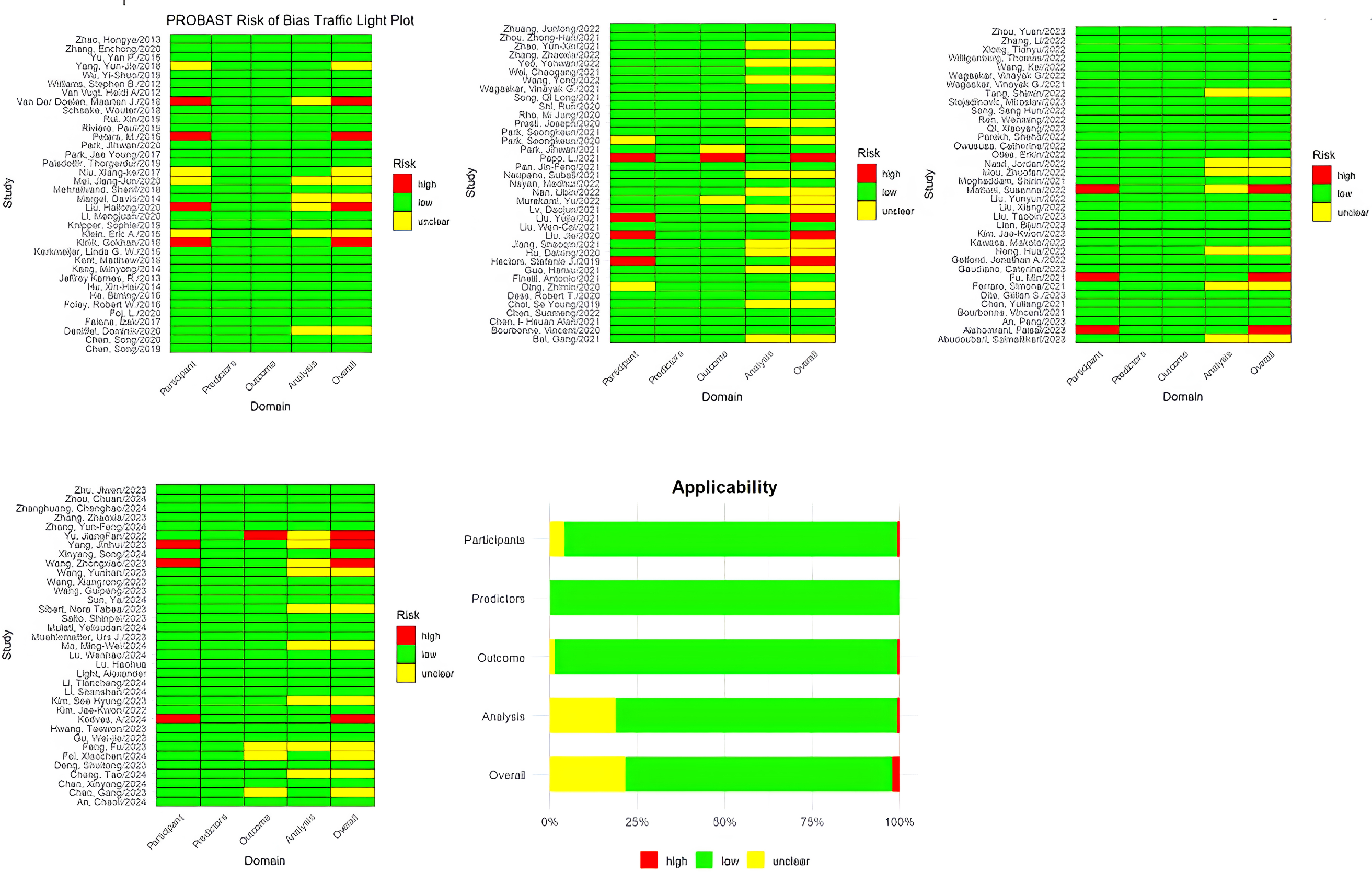

Supplement: Supplementary file 1 [file Image1.tif]

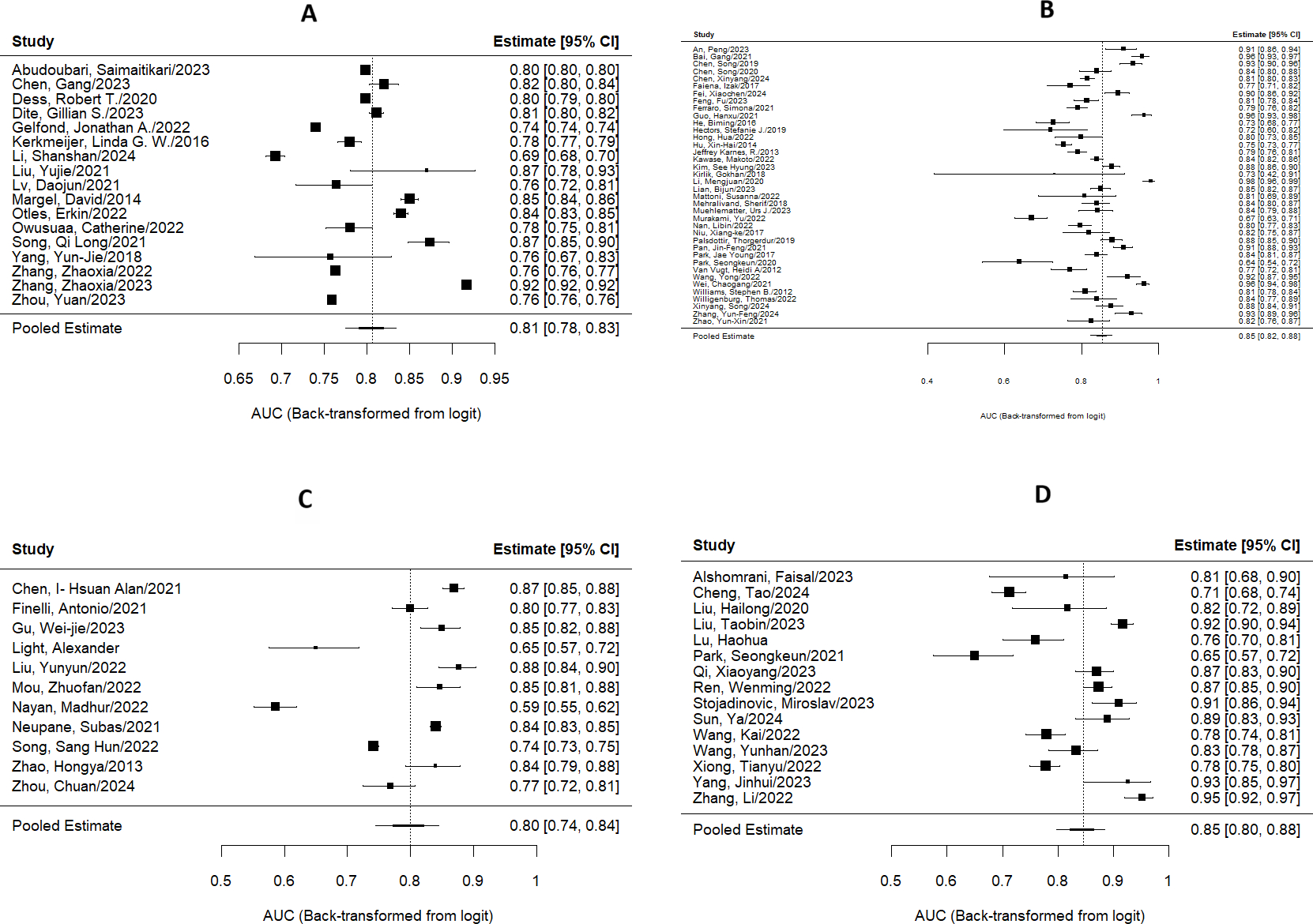

Supplement: Supplementary file 2 [file Image2.tif]
