# Supplementary material for: Prediction models in prostate cancer: a systematic review and meta-analysis
Source: Front Oncol. 2026 May 1;16:1705780. doi: 10.3389/fonc.2026.1705780 (PMC13175879; doi:10.3389/fonc.2026.1705780)
Supplement: Supplementary file 4 [file Table2.docx]

|  |  | | S2 Table. Total performance score of included studies | | | | | | | | | | | | | |
| --- | --- | --- | --- | --- | --- | --- | --- | --- | --- | --- | --- | --- | --- | --- | --- | --- |
| **Author** | | **Sample  size** | | **Feature used** | **Multicenter  study** | | **Disease stage** | **Histology** | **Event Rate** | **Treatment** | **Model used** | **FST** | **MS** | | **Validation** | **Overall  score** |
| Wang G et al(82). | | 3 | | 4 | 0 | 1 | | 1 | 0 | 1 | 1 | | 0 | 2 | 1 | 14 |
| Kedves, A et al(69) | | 1 | | 1 | 0 | 1 | | 1 | 0 | 1 | 2 | | 0 | 1 | 2 | 10 |
| Chen X et al(83). | | 5 | | 1 | 0 | 1 | | 1 | 0 | 0 | 2 | | 1 | 2 | 1 | 14 |
| Muehlematter U et al(73) | | 3 | | 1 | 0 | 1 | | 1 | 0 | 0 | 1 | | 0 | 3 | 4 | 14 |
| bFei, X et al(84) | | 3 | | 2 | 0 | 1 | | 1 | 0 | 0 | 2 | | 2 | 2 | 4 | 17 |
| Light, A et al(85) | | 2 | | 2 | 2 | 1 | | 1 | 0 | 0 | 3 | | 0 | 2 | 4 | 17 |
| Chen, G et al(86) | | 5 | | 2 | 0 | 1 | | 1 | 0 | 0 | 1 | | 1 | 2 | 1 | 14 |
| Feng, F et al(87) | | 4 | | 1 | 0 | 1 | | 1 | 0 | 0 | 1 | | 1 | 1 | 1 | 11 |
| Xinyang, S et al(65) . | | 3 | | 2 | 2 | 1 | | 1 | 0 | 0 | 3 | | 2 | 3 | 4 | 21 |
| Wang, Z et al(88) | | 1 | | 2 | 0 | 1 | | 1 | 0 | 0 | 2 | | 0 | 1 | 2 | 9 |
| Saito, S et al(89) | | 3 | | 1 | 0 | 1 | | 1 | 0 | 1 | 2 | | 0 | 1 | 1 | 11 |
| Cheng, T et al(90) | | 4 | | 2 | 1 | 1 | | 1 | 0 | 0 | 2 | | 1 | 2 | 2 | 15 |
| Hwang, T et al(91) | | 5 | | 3 | 0 | 1 | | 1 | 0 | 0 | 1 | | 0 | 2 | 2 | 15 |
| Sun, Y et al(92) | | 2 | | 2 | 0 | 1 | | 1 | 0 | 0 | 3 | | 2 | 3 | 2 | 16 |
| Zhang, Z et al(93) | | 5 | | 3 | 3 | 1 | | 1 | 0 | 0 | 1 | | 2 | 3 | 1 | 20 |
| Deng, S et al(94) | | 3 | | 1 | 2 | 1 | | 1 | 0 | 0 | 3 | | 0 | 3 | 4 | 18 |
| Lu, W et al(66) | | 4 | | 1 | 0 | 1 | | 1 | 0 | 0 | 3 | | 1 | 3 | 1 | 15 |
| Huiling L et al(95) | | 3 | | 2 | 0 | 1 | | 1 | 0 | 0 | 1 | | 2 | 3 | 4 | 17 |
| Sibert, N et al(96) | | 5 | | 2 | 1 | 1 | | 1 | 0 | 0 | 1 | | 2 | 3 | 4 | 20 |
| Mulati, Y et al(97) | | 3 | | 2 | 2 | 1 | | 1 | 0 | 0 | 1 | | 2 | 2 | 1 | 15 |
| Wang, X et al(38) | | 5 | | 2 | 2 | 1 | | 1 | 0 | 0 | 3 | | 1 | 3 | 4 | 22 |
| Yang, J et al(98) | | 1 | | 1 | 0 | 1 | | 1 | 0 | 0 | 1 | | 1 | 2 | 2 | 9 |
| Ma, M et al(99) | | 3 | | 1 | 0 | 1 | | 1 | 0 | 0 | 1 | | 2 | 2 | 2 | 12 |
| An, C et al(100) | | 2 | | 1 | 0 | 1 | | 1 | 0 | 0 | 1 | | 2 | 2 | 2 | 12 |
| Gu, W et al(101) | | 4 | | 2 | 3 | 1 | | 1 | 0 | 0 | 2 | | 0 | 2 | 4 | 19 |
| Kim, SH et al(44) | | 4 | | 3 | 0 | 1 | | 1 | 0 | 0 | 2 | | 1 | 3 | 2 | 16 |
| Zhang, Y et al(45) | | 3 | | 2 | 0 | 1 | | 1 | 0 | 0 | 3 | | 2 | 2 | 2 | 16 |
| Zhu, J et al(102) | | 4 | | 2 | 2 | 1 | | 1 | 0 | 0 | 1 | | 1 | 2 | 2 | 16 |
| Li, S et al(103) | | 5 | | 2 | 2 | 1 | | 1 | 1 | 0 | 1 | | 1 | 2 | 1 | 17 |
| Wang, Y et al(104) | | 3 | | 2 | 0 | 1 | | 1 | 1 | 0 | **2** | | 2 | 3 | 2 | 17 |
| Lu, H et al(56) | | 3 | | 2 | 0 | 1 | | 1 | 0 | 0 | 2 | | 1 | 2 | 2 | 14 |
| Zhanghuang, C et al(105) | | 5 | | 2 | 2 | 1 | | 1 | 0 | 0 | 1 | | 2 | 3 | 4 | 21 |
| Zhou, C et al(61) | | 3 | | 2 | 3 | 1 | | 1 | 0 | 0 | 2 | | 1 | 3 | 4 | 20 |
| Kim, J et al(46) | | 3 | | 2 | 2 | 1 | | 1 | 0 | 0 | 3 | | 0 | 2 | 2 | 16 |
| Yu, J et al(106) | | 3 | | 1 | 0 | 1 | | 1 | 0 | 1 | 1 | | 1 | 2 | 2 | 12 |
| Mou, Z et al(107) | | 3 | | 2 | 0 | 1 | | 1 | 0 | 0 | 2 | | 1 | 2 | 2 | 13 |
| Owusuaa, C et al(108) | | 4 | | 2 | 3 | 1 | | 1 | 1 | 0 | 1 | | 0 | 3 | 4 | 20 |
| Song, S et al(62) | | 5 | | 1 | 2 | 1 | | 1 | 1 | 0 | 2 | | 1 | 3 | 4 | 21 |
| Liu, Y et al(109) | | 3 | | 2 | 0 | 1 | | 1 | 1 | 0 | 1 | | 2 | 2 | 1 | 14 |
| Lian, B et al(110) | | 4 | | 2 | 1 | 1 | | 1 | 0 | 0 | 1 | | 1 | 3 | 4 | 18 |
| Ren, W et al(111) | | 4 | | 2 | 0 | 1 | | 1 | 0 | 0 | 1 | | 2 | 2 | 1 | 14 |
| Stojadinovic, M et al(112) | | 3 | | 2 | 0 | 1 | | 1 | 1 | 0 | 1 | | 0 | 2 | 1 | 12 |
| Gaudiano, C et al(68) | | 2 | | 2 | 0 | 1 | | 1 | 0 | 0 | 1 | | 2 | 3 | 4 | 16 |
| Zhang, L et al(113) | | 3 | | 2 | 0 | 1 | | 1 | 0 | 0 | 2 | | 2 | 2 | 1 | 14 |
| Abudoubari, S et al(114) | | 5 | | 1 | 1 | 1 | | 1 | 0 | 0 | 1 | | 2 | 3 | 2 | 16 |
| Wang, K et al(70) | | 4 | | 2 | 0 | 1 | | 1 | 0 | 0 | 2 | | 2 | 2 | 2 | 16 |
| Liu, X et al(115) | | 5 | | 2 | 1 | 1 | | 1 | 0 | 0 | 1 | | 2 | 1 | 1 | 15 |
| Alshomrani, F et al(116) | | 1 | | 2 | 0 | 1 | | 1 | 0 | 0 | 1 | | 0 | 2 | 2 | 10 |
| Gelfond, J et al(117) | | 5 | | 2 | 1 | 1 | | 1 | 0 | 0 | 1 | | 1 | 3 | 4 | 16 |
| Dite, G et al(118) | | 5 | | 2 | 1 | 1 | | 1 | 0 | 0 | 1 | | 1 | 2 | 1 | 15 |
| Qi, X et al(71) | | 3 | | 2 | 0 | 1 | | 1 | 0 | 0 | 3 | | 2 | 2 | 2 | 16 |
| Zhou, Y et al(119) | | 5 | | 2 | 1 | 1 | | 1 | 0 | 0 | 1 | | 2 | 3 | 4 | 20 |
| Nasri, J et al(120) | | 3 | | 3 | 0 | 1 | | 1 | 0 | 0 | 1 | | 1 | 3 | 2 | 14 |
| Liu, T et al(57) | | 4 | | 2 | 0 | 1 | | 1 | 0 | 0 | 2 | | 2 | 2 | 2 | 16 |
| Mattoni, S et al(121) | | 1 | | 2 | 3 | 1 | | 1 | 0 | 0 | 1 | | 1 | 2 | 2 | 13 |
| An, P et al(122) | | 3 | | 3 | 0 | 1 | | 1 | 0 | 0 | 1 | | 1 | 3 | 4 | 17 |
| Kawase, M et al(47) | | 5 | | 2 | 1 | 1 | | 1 | 0 | 0 | 2 | | 1 | 2 | 2 | 17 |
| Kim, J et al(123) | | 5 | | 2 | 1 | 1 | | 1 | 0 | 0 | 3 | | 0 | 2 | 2 | 17 |
| Willigenburg, T et al(124) | | 2 | | 3 | 0 | 1 | | 1 | 0 | 1 | 2 | | 0 | 2 | 2 | 14 |
| Moghaddam, S et al(125) | | 4 | | 2 | 2 | 1 | | 1 | 0 | 0 | 3 | | 1 | 3 | 4 | 21 |
| Tang, S et al(126) | | 3 | | 2 | 0 | 1 | | 1 | 0 | 0 | 1 | | 2 | 2 | 2 | 13 |
| Chen, Y et al(48) | | 5 | | 2 | 0 | 1 | | 1 | 0 | 0 | 2 | | 2 | 3 | 4 | 20 |
| Otles, E et al(127) | | 5 | | 2 | 1 | 1 | | 1 | 0 | 0 | 2 | | 0 | 3 | 4 | 19 |
| Xiong, T et al(128) | | 4 | | 3 | 0 | 1 | | 1 | 0 | 0 | 1 | | 1 | 2 | 1 | 14 |
| Bourbonne, V et al(129) | | 3 | | 2 | 0 | 1 | | 1 | 0 | 0 | 2 | | 0 | 2 | 1 | 12 |
| Parekh, S et al(130) | | 5 | | 2 | 0 | 1 | | 1 | 0 | 0 | 1 | | 2 | 3 | 4 | 19 |
| Ferraro, S et al(49) | | 4 | | 2 | 0 | 1 | | 1 | 0 | 0 | 1 | | 0 | 3 | 2 | 13 |
| Wagaskar, V et al(131) | | 4 | | 2 | 0 | 1 | | 1 | 0 | 0 | 1 | | 0 | 3 | 4 | 16 |
| Fu, M et al(132) | | 1 | | 2 | 1 | 1 | | 1 | 0 | 0 | 2 | | 2 | 3 | 4 | 17 |
| Hong, H et al(50) | | 2 | | 2 | 0 | 1 | | 1 | 0 | 0 | 1 | | 1 | 2 | 2 | 11 |
| Wagaskar, V et al(133) | | 5 | | 2 | 0 | 1 | | 1 | 0 | 0 | 1 | | 1 | 3 | 4 | 18 |
| Yeo, Y et al(134) | | 5 | | 2 | 1 | 1 | | 1 | 0 | 0 | 1 | | 0 | 2 | 2 | 14 |
| Jiang, S et al(29) | | 4 | | 2 | 0 | 1 | | 1 | 0 | 0 | 3 | | 2 | 2 | 2 | 16 |
| Zhang, Z et al(135) | | 5 | | 2 | 1 | 1 | | 1 | 0 | 0 | 1 | | 2 | 1 | 1 | 15 |
| Chen, S et al(52) | | 4 | | 1 | 0 | 1 | | 1 | 0 | 0 | 3 | | 1 | 1 | 2 | 14 |
| Murakami, Y et al(136) | | 3 | | 2 | 0 | 1 | | 1 | 0 | 0 | 2 | | 0 | 2 | 4 | 15 |
| Song, QL et al(60) | | 4 | | 1 | 0 | 1 | | 1 | 0 | 0 | 1 | | 2 | 1 | 1 | 12 |
| Nan, L et al(43) | | 4 | | 2 | 0 | 1 | | 1 | 0 | 0 | 1 | | 2 | 2 | 2 | 14 |
| Nayan, M et al(54) | | 4 | | 2 | 0 | 1 | | 1 | 0 | 0 | 2 | | 0 | 1 | 2 | 13 |
| Zhuang, J et al(30) | | 4 | | 3 | 1 | 1 | | 1 | 0 | 0 | 2 | | 0 | 2 | 2 | 16 |
| Pan, J et al(137) | | 4 | | 2 | 0 | 1 | | 1 | 0 | 0 | 1 | | 1 | 1 | 1 | 12 |
| Wang, Y et al(138) | | 2 | | 2 | 0 | 1 | | 1 | 0 | 0 | 2 | | 1 | 3 | 2 | 13 |
| Liu, Y et al(139) | | 1 | | 1 | 0 | 1 | | 1 | 0 | 0 | 1 | | 2 | 2 | 2 | 11 |
| Chen, I et al(55) | | 5 | | 1 | 0 | 1 | | 1 | 0 | 0 | 1 | | 0 | 2 | 2 | 13 |
| Papp, L et al(140) | | 1 | | 2 | 0 |  | | 1 | 0 | 0 | 1 | | 0 | 2 | 2 | 10 |
| Park, J et al(141) | | 5 | | 2 | 2 | 1 | | 1 | 0 | 0 | 1 | | 0 | 2 | 2 | 16 |
| Park, S et al(58) | | 2 | | 2 | 0 | 1 | | 1 | 0 | 0 | 2 | | 0 | 2 | 2 | 12 |
| Bai, G et al(142) | | 3 | | 2 | 0 | 1 | | 1 | 0 | 0 | 1 | | 1 | 2 | 2 | 12 |
| Wagaskar, V et al(131) | | 2 | | 2 | 0 | 1 | | 1 | 0 | 0 | 1 | | 0 | 2 | 1 | 10 |
| Ding, Z et al(31) | | 2 | | 1 | 0 | 1 | | 1 | 0 | 0 | 2 | | 1 | 1 | 2 | 11 |
| Liu, W et al(32) | | 5 | | 2 | 1 | 1 | | 1 | 0 | 0 | 3 | | 2 | 1 | 2 | 18 |
| Dess, R et al(143) | | 5 | | 2 | 1 | 1 | | 1 | 0 | 0 | 2 | | 0 | 3 | 4 | 19 |
| Guo, H et al(33) | | 3 | | 2 | 1 | 1 | | 1 | 0 | 0 | 2 | | 0 | 2 | 2 | 13 |
| Zhao, Y et al(144) | | 2 | | 2 | 0 | 1 | | 1 | 0 | 1 | 1 | | 2 | 2 | 2 | 13 |
| Lv, D et al(145) | | 3 | | 2 | 1 | 1 | | 1 | 0 | 0 | 1 | | 2 | 2 | 2 | 14 |
| Hu, D et al(146) | | 4 | | 2 | 1 | 1 | | 1 | 0 | 0 | 1 | | 2 | 2 | 2 | 15 |
| Neupane, S et al(147) | | 5 | | 2 | 0 | 1 | | 1 | 0 | 0 | 1 | | 0 | 2 | 2 | 13 |
| Rho, MJ et al(34) | | 5 | | 1 | 3 | 1 | | 1 | 0 | 0 | 2 | | 0 | 2 | 2 | 17 |
| Liu, J et al(148) | | 1 | | 1 | 0 | 1 | | 1 | 1 | 0 | 1 | | 0 | 2 | 2 | 10 |
| Wei, Cet al(149) | | 4 | | 2 | 0 | 1 | | 1 | 1 | 0 | 1 | | 1 | 2 | 2 | 15 |
| Bourbonne, V et al(150) | | 2 | | 2 | 2 | 1 | | 1 | 0 | 0 | 1 | | 0 | 3 | 4 | 16 |
| Park, S et al(72) | | 2 | | 2 | 0 | 1 | | 1 | 1 | 0 | 3 | | 2 | 3 | 4 | 19 |
| Zhou, Z et al(51) | | 5 | | 1 | 2 | 1 | | 1 | 1 | 0 | 1 | | 2 | 3 | 4 | 21 |
| Presti, J et al(28) | | 5 | | 2 | 1 | 1 | | 1 | 1 | 0 | 2 | | 2 | 2 | 2 | 18 |
| Finelli, A et l(151) | | 4 | | 2 | 1 | 1 | | 1 | 1 | 1 | 1 | | 1 | 2 | 2 | 17 |
| Hectors, S et al(35) | | 1 | | 2 | 0 | 1 | | 1 | 1 | 0 | 1 | | 2 | 2 | 2 | 13 |
| Choi, SY et al(152) | | 4 | | 1 | 1 | 1 | | 1 | 1 | 1 | 1 | | 1 | 2 | 2 | 15 |
| Shi, R et al(153) | | 4 | | 1 | 3 | 1 | | 1 | 1 | 0 | 1 | | 1 | 3 | 4 | 20 |
| Wu, Y et al | | 4 | | 1 | 2 | 1 | | 1 | 0 | 0 | 2 | | 1 | 3 | 4 | 19 |
| Li, M et al(36) | | 3 | | 2 | 0 | 1 | | 1 | 0 | 0 | 1 | | 2 | 2 | 1 | 13 |
| Chen, S et al(154) | | 3 | | 1 | 0 | 1 | | 1 | 0 | 0 | 2 | | 2 | 2 | 1 | 13 |
| Rui, X et al(155) | | 3 | | 1 | 0 | 1 | | 1 | 0 | 0 | 1 | | 1 | 2 | 2 | 12 |
| Riviere, P et al(156) | | 5 | | 2 | 1 | 1 | | 1 | 0 | 0 | 1 | | 2 | 2 | 2 | 17 |
| Zhang, E et al(157) | | 3 | | 2 | 0 | 1 | | 1 | 0 | 0 | 1 | | 2 | 2 | 2 | 14 |
| Foj, L et al(158) | | 3 | | 2 | 0 | 1 | | 1 | 0 | 0 | 1 | | 0 | 2 | 1 | 11 |
| Palsdottir, T et al(39) | | 4 | | 1 | 1 | 1 | | 1 | 0 | 0 | 1 | | 0 | 2 | 1 | 12 |
| Knipper, S et al(159) | | 5 | | 2 | 0 | 1 | | 1 | 0 | 0 | 1 | | 0 | 3 | 4 | 17 |
| Mei, J et al(42) | | 2 | | 2 | 0 | 1 | | 1 | 0 | 0 | 1 | | 1 | 2 | 2 | 11 |
| Kirlik, G et al(160) | | 1 | | 1 | 0 | 1 | | 1 | 0 | 0 | 2 | | 0 | 2 | 2 | 10 |
| Liu, H et al(59) | | 1 | | 1 | 0 | 1 | | 1 | 0 | 0 | 1 | | 2 | 2 | 2 | 10 |
| Park, J et al(64) | | 5 | | 2 | 0 | 1 | | 1 | 0 | 0 | 2 | | 0 | 2 | 2 | 15 |
| Deniffel, D et al(161) | | 4 | | 2 | 0 | 1 | | 1 | 0 | 0 | 1 | | 2 | 2 | 2 | 14 |
| Chen, S et al(37) | | 3 | | 2 | 1 | 1 | | 1 | 0 | 0 | 1 | | 2 | 2 | 4 | 17 |
| Peters, M et al(162) | | 1 | | 2 | 1 | 1 | | 1 | 0 | 1 | 1 | | 2 | 1 | 1 | 12 |
| Van D et al(163) | | 1 | | 1 | 0 | 1 | | 1 | 0 | 1 | 1 | | 2 | 2 | 2 | 11 |
| Yang, Y et al(74) | | 2 | | 1 | 0 | 1 | | 1 | 0 | 1 | 1 | | 2 | 3 | 4 | 16 |
| Yu, YP et al(164) | | 3 | | 2 | 0 | 1 | | 1 | 0 | 0 | 1 | | 0 | 2 | 2 | 12 |
| Kerkmeijer, LGW et al(165) | | 5 | | 1 | 3 | 1 | | 1 | 0 | 1 | 1 | | 0 | 2 | 2 | 17 |
| Niu, X et al(166) | | 2 | | 1 | 0 | 1 | | 1 | 0 | 0 | 1 | | 2 | 2 | 1 | 11 |
| He, B et al(167) | | 3 | | 2 | 0 | 1 | | 1 | 0 | 0 | 1 | | 2 | 1 | 1 | 12 |
| Mehralivand, S et al(40) | | 3 | | 1 | 0 | 1 | | 1 | 0 | 0 | 1 | | 2 | 3 | 4 | 16 |
| Foley, RW et al(168) | | 3 | | 2 | 1 | 1 | | 1 | 0 | 0 | 3 | | 2 | 2 | 2 | 17 |
| Faiena, I et al(169) | | 3 | | 2 | 0 | 1 | | 1 | 0 | 0 | 1 | | 2 | 2 | 1 | 13 |
| Kent, M et al(170) | | 5 | | 2 | 3 | 1 | | 1 | 0 | 0 | 1 | | 0 | 3 | 4 | 20 |
| Park, JY et al(41) | | 4 | | 1 | 0 | 1 | | 1 | 0 | 0 | 1 | | 1 | 3 | 4 | 16 |
| Schaake, W et al(171) | | 3 | | 1 | 0 | 1 | | 1 | 0 | 1 | 1 | | 1 | 2 | 1 | 12 |
| Klein, EA et al(172) | | 2 | | 2 | 0 | 1 | | 1 | 0 | 0 | 1 | | 2 | 2 | 2 | 12 |
| Kang, M et al(173) | | 5 | | 2 | 0 | 1 | | 1 | 0 | 0 | 1 | | 0 | 2 | 1 | 13 |
| Van V et al(174) | | 3 | | 1 | 0 | 1 | | 1 | 0 | 0 | 1 | | 0 | 3 | 4 | 14 |
| Hu, X et al(63) | | 5 | | 2 | 0 | 1 | | 1 | 0 | 0 | 3 | | 0 | 2 | 1 | 15 |
| Williams, SB et al(175) | | 4 | | 2 | 1 | 1 | | 1 | 0 | 0 | 1 | | 2 | 3 | 4 | 19 |
| Margel, D et al(176) | | 5 | | 2 | 0 | 1 | | 1 | 0 | 0 | 1 | | 0 | 3 | 2 | 14 |
| Jeffrey KR et al(177) | | 4 | | 2 | 0 | 1 | | 1 | 0 | 0 | 2 | | 2 | 3 | 4 | 19 |
| Zhao, H et al(53) | | 3 | | 1 | 0 | 1 | | 1 | 0 | 0 | 3 | | 0 | 2 | 2 | 13 |

| **Scoring system used** | **Possible maximum score: 27** |  |  |
| --- | --- | --- | --- |
| **Sample size (SS)**  SS ≤ 100 = 1  100 < SS ≤ 200 = 2  200 < SS ≤ 500 = 3  500 < SS ≤ 1000 = 4  SS > 1000 = 5  **Disease Stage**  No =0  Yes =1  **Treatment**  No =0  Yes =1 | **Feature Used**  Clinical only =1  Pathological only =1  Imaging only =1  Radiomics only =1  **Multi-center study**  Yes = 2 (2 centers), 3(≥3 centers), 4 (≥3 centers with various countries)  No = 0  **Feature selection Technique (FST)**  Not stated =0  1used = 1  >1used = 2 | **Prediction Algorithm (AI)**  1AI & 1 model =1  1AI & >1 model =2  >1AI & >1 model =3  **Model Assessment (MS)**  Validation accuracy without confidence interval (CI) =1  Validation accuracy with CI =2  Train and validation accuracy with CI =3  **Histology**  No=0  Yes=1 | **Model Validation:**  Train-test model validation = 1, Bootstrap validation/cross validation =2,  External validation =4  Event Rate  No=0  Yes=1 |
