# Supplementary material for: Prediction models in prostate cancer: a systematic review and meta-analysis
Source: Front Oncol. 2026 May 1;16:1705780. doi: 10.3389/fonc.2026.1705780 (PMC13175879; doi:10.3389/fonc.2026.1705780)
Supplement: Supplementary file 5 [file Table3.docx]

Supplementary Table S3. Full Search Strategy

| **Database** | **Search Fields** | **Search Query** |
| --- | --- | --- |
| PubMed | Title, Abstract, MeSH | (machine AND learning AND model AND for AND prostate AND cancer AND prediction) OR ("Cancer of the prostate") OR ("Prostate cancer") OR ("Prostate Tumors") AND ("Risk model") OR ("Risk assessment model") OR ("Risk prediction model") OR ("Assessment tool") OR ("Prediction score"). Further modified for specificity to (("Cancer of the prostate") OR ("prostate cancer") OR ("prostate Tumors")) AND (("Risk prediction Model") OR ("prediction model")).  Further modified for specificity to (("Cancer of the prostate") OR ("prostate cancer") OR ("prostate Tumors")) AND (("Risk prediction Model") OR ("prediction model")). |
| Scopus | TITLE-ABS-KEY | TITLE-ABS-KEY (machine AND learning AND model AND for AND prostate AND cancer AND prediction) OR ("Cancer of the prostate") AND TITLE-ABS-KEY ("Prostate cancer" OR "Prostate Tumors") AND TITLE-ABS-KEY ("Risk model" OR "Risk assessment model" OR "Risk prediction model" OR "Assessment tool" OR "Prediction score").  Further modified for specificity to TITLE-ABS-KEY ("Cancer of the prostate" OR "prostate cancer") OR ("prostate Tumors") AND TITLE-ABS-KEY ("Risk prediction Model") OR ("prediction model"). |

The study constructed search keywords, which were used to execute search queries to identify relevant articles from the databases. The search was carried out on October 17, 2024. No language restriction was applied, but articles were search from 2012-2024.
